# Targeted next-generation sequencing identifies *ABCA4* mutations in Chinese families with childhood-onset and adult-onset Stargardt disease

**DOI:** 10.1042/BSR20203497

**Published:** 2021-06-02

**Authors:** Ling-hui Qu, Xin Jin, Chao Zeng, Nian-gou Zhou, Yan-hong Liu, Ye Lin

**Affiliations:** 1 Department of Ophthalmology, The 74th Army Group Hospital, Guangzhou 510318, China; 2 Department of Ophthalmology, General Hospital of Chinese People’s Liberation Army, Beijing 100853, China

**Keywords:** ABCA4 gene, childhood-onset, Mutation detection, Next-generation sequencing, Stargardt disease

## Abstract

**Background**: Stargardt disease (STGD) is the most common form of juvenile macular dystrophy associated with progressive central vision loss, and is agenetically and clinically heterogeneous disease. Molecular diagnosis is of great significance in aiding the clinical diagnosis, helping to determine the phenotypic severity and visual prognosis. In the present study, we determined the clinical and genetic features of seven childhood-onset and three adult-onset Chinese STGD families. We performed capture next-generation sequencing (NGS) of the probands and searched for potentially disease-causing genetic variants in previously identified retinal or macular dystrophy genes.

**Methods**: In all, ten unrelated Chinese families were enrolled. Panel-based NGS was performed to identify potentially disease-causing genetic variants in previously identified retinal or macular dystrophy genes, including the five known STGD genes (*ABCA4, PROM1, PRPH2, VMD2*, and *ELOVL4*). Variant analysis, Sanger validation, and segregation tests were utilized to validate the disease-causing mutations in these families.

**Results:** Using systematic data analysis with an established bioinformatics pipeline and segregation analysis, 17 pathogenic mutations in *ABCA4* were identified in the 10 STGD families. Four of these mutations were novel: c.371delG, c.681T > G, c.5509C > T, and EX37del. Childhood-onset STGD was associated with severe visual loss, generalized retinal dysfunction and was due to more severe variants in *ABCA4* than those found in adult-onset disease.

**Conclusions:** We expand the existing spectrum of STGD and reveal the genotype–phenotype relationships of the *ABCA4* mutations in Chinese patients. Childhood-onset STGD lies at the severe end of the spectrum of *ABCA4*-associated retinal phenotypes.

## Introduction

Stargardt disease (STGD; MIM 248200) is the most common inherited juvenile onset macular dystrophy, with a prevalence of approximately 1:8000 to 1:10000, depending on the population studied [1,2]. It is characterized by a decrease in central vision and the presence of bilateral atrophic-appearing foveal lesions. These lesions may have a beaten metal appearance with or without yellowish-white fundus flecks at the posterior pole or mid-peripheral retina.

STGD is mostly inherited in autosomal recessive mode, although an autosomal dominant form has been also reported [3]. Mutations in *ABCA4* (MIM 601691; transcript number ENSP 00000359245.3), also known as *ABCR*, are responsible for most cases with autosomal recessive STGD, and are also implicated in other retinal degenerative diseases such as retinitis pigmentosa type 19, cone-rod dystrophy, and age-related macular degeneration [3,4]. To date, more than 1200 disease-causing mutations have been reported in *ABCA4* [5]. Rare cases of STGD or ‘STGD-like’ disease phenotypes have been reported with mutations in *PRPH2, VMD2, ELOVL4*, and *PROM1* genes with important roles in maintaining physiological macular function [3,6–8]. Moreover, it should be noted that STGD or ‘STGD-like’ disease presents with highly variable phenotypes and progression [3,6–8]. Clinical features as well as onset and progression of disease can be highly variable.

STGD is particularly devastating because affected individuals lose childhood central vision, which is necessary for common tasks including reading, schooling, driving, and recognizing faces. Ongoing stem cell-based therapy [9,10] or gene therapy [11] has paved the way to clinical trials for the treatment of STGD. Therefore, accurate and comprehensive molecular diagnosis is critical as an aid to clinical diagnosis, determining the visual prognosis, offering a basis for novel therapeutic approaches, and is also crucial for prenatal diagnosis. However, the high genetic heterogeneity in STGD makes molecular genetic analyses very challenging.

Numerous other recent papers have reported the molecular clinical characteristics and have described longitudinal studies of STGD or STGD-like disease in Britain [12–16], the United States [17], Canada [18], Mexico [19], Spain [2], the Netherlands [20,21], Denmark [22], Brazil [23], and other American and European areas. Moreover, the international multicentre ‘Natural History of the Progression of Atrophy Secondary to Stargardt Disease (ProgStar)’ project has led the largest genetic and clinical studies in this research area [24,25]. In addition, studies have increasingly reported on the molecular and clinical characteristics of STGD in Chinese patients [7,8,26–32]. The mutation spectrum of Chinese STGD patients is reportedly quite different from that observed in other populations, with results suggesting that most of the *ABCA4* mutations may be unique to Chinese patients. For example, the nonsense mutation p.Tyr808Ter was the most common mutation in Chinese STGD (with a frequency of 4.7%), but was not observed in patients of any other ethnicity [28]. However, genetic and clinical correlations between childhood-onset and adult-onset Stargardt patients in the Chinese population have not been elucidated.

In the present study, we utilized targeted exome sequencing (TES) to investigate genetic anomalies in ten Chinese STGD families and found four novel mutations. These variants segregated with the diverse disease phenotypes in the respective families and were not detected in the healthy controls. We also identified genotype–phenotype correlations between these *ABCA4* mutations, and compared the molecular and clinical characteristics of childhood-onset STGD patients with those of adult-onset patients, to look for predictors of long-term visual prognosis and indicators of possible treatment approaches.

## Materials and methods

### Pedigrees and controls

**Figure 1:**
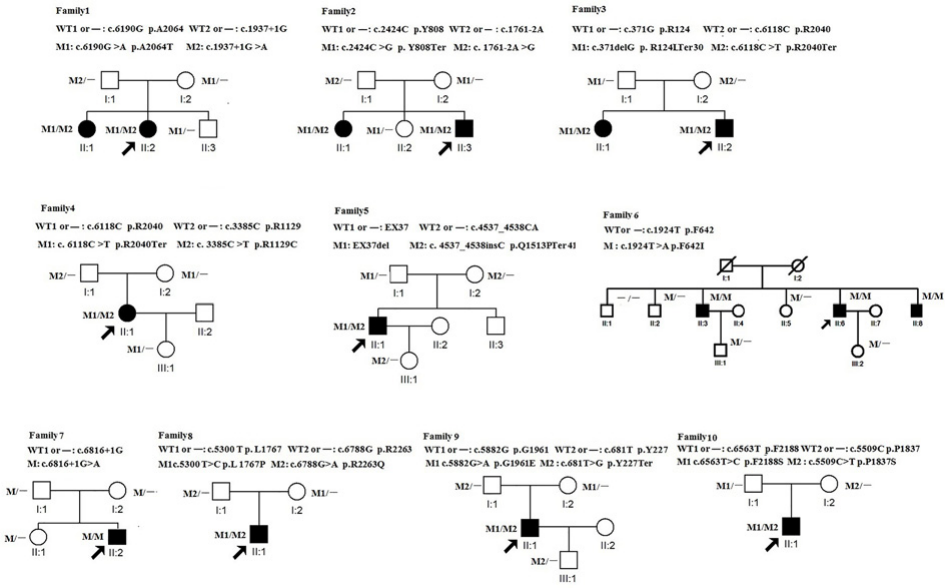
Pedigrees of families with STGD Squares and circles indicate men and women, respectively, and dark symbols represent the affected individuals. The probands are indicated by an arrow. –, wildtype; M, mutation.

The present study adhered to the tenets of the Declaration of Helsinki. The protocol was approved by the Ethics Committee of the 74^th^ Army Group Hospital, Guangzhou, China. Written informed consent was obtained from all participants. Peripheral blood samples were collected from both the affected patients and unaffected relatives, and detailed family histories were obtained from interviews with the patients and relatives (Figure 1). The age of onset was defined as the age at which visual loss was first noted by the patient. We defined ‘childhood-onset Stargardt’ at the onset of <17 years of age and classified adult-onset as ≥17 years [14,15].

Comprehensive ophthalmic examinations included best-corrected visual acuity (BCVA) assessment, dilated fundus examination, color fundus photography (TRC-NW8F plus, TOPCON, Tokyo, Japan), fundus autofluorescence imaging (FAF; excitation light: 488 nm, barrier filter: 500 nm; Spectralis, Heidelberg Engineering, Heidelberg, Germany), and spectral-domain optical coherence tomography (SD-OCT; Spectralis, Heidelberg Engineering). Electrophysiological assessment included full-field electroretinography and pattern electroretinography (FERG and PERG, respectively; Diagnosys LLC, Lowell, MA), and was conducted in accordance with the standards of the International Society for Clinical Electrophysiology of Vision [12,15,16]. PERG P50 was used to measure macular function; cone function was assessed using light-adapted 30 Hz and light-adapted 3.0 ERG protocols, and rod ERG abnormality was assessed using dark-adapted 0.01 and dark-adapted 10.0 ERG protocols. All the components of the ERG and the PERG P50 component were examined to classify patients into one of the three previously described groups (Supplementary Table S1) [12,15,16]: Group 1 was defined as PERG abnormality with normal FERGs. Group 2 showed PERG abnormality andabnormal cone function (assessed with light-adapted 30 Hz and light-adapted 3.0) on FERG. In Group 3, additional rod ERG abnormality (assessed using dark-adapted 0.01 and dark-adapted 10.0) was found. The overall classification was based on the more severely affected eye in the small number of patients with an interocular difference in ERG group.

Patients were also classified into three fundus appearance groups based on the presence and location of central (macular) retinal pigment epithelium (RPE) atrophy and yellowish-white flecks according to a previous report (Supplementary Table S1) [13,14]. The FAF phenotype was also classified into three patterns as previously described (Supplementary Table S1) [13,14].

The classification of severity of the STGD phenotype was made on the basis of the clinical findings (Supplementary Table S2): age of onset, BCVA, fundus appearance, FAF pattern, and electrophysiologic grouping [13,14].

Genotype was classified into three groups based on the number and type of the identified variants, as previously described (Supplementary Table S1) [13,14,24]. Null variants were those that would be expected to affect splicing or introduce a premature truncating codon in the protein if translated.

A group of 200 matching control individuals was also recruited by advertisement, with no symptoms of visual impairment and no personal or family history of inherited disease. All ten pedigrees and control individuals described in the present study were from South and Southwest China.

### Targeted region capture and next-generation sequencing

A sequence capture array (Roche NimbleGen, Madison, WI), from Beijing Genomics Institute-ShenZhen (BGI, Inc., Shenzhen, China), was used to capture the coded exons of the 103 hereditary retinal diseases’ genes, including *ABCA4* and another four known implicated STGD genes (*PROM1*,*PRPH2, VMD2*, and *ELOVL4*). The genomic DNA of the proband was extracted from peripheral blood according to the manufacturer’s standard procedure using the QIAamp DNA Blood Midi Kit (Qiagen, Hilden, Germany) using our previously described approach [33–36].

### Filtering procedures of detected variants

All captured libraries were sequenced using the Illumina HiSeq 2000 platform (paired-end, average depth ≥ 100×). To detect the potential pathogenic variants of the probands, we used filtering criteria to generate clean reads (with a length of 90 bp) for further analysis, and then aligned the clean reads against the human genome reference from the National Center for Biotechnology Information (NCBI) database (version hg19; http://www.ncbi.nlm.nih.gov/projects/genome/assembly/grc/) using the Burrows Wheeler Aligner Multi-Vision software package (http://bio-bwa.sourceforge.net/). Single-nucleotide variants and Indels were identified using SOAPsnp software and Samtools Indel Genotyper (http://samtools.sourceforge.net/), respectively. All single-nucleotide variants and Indels were determined using the NCBI dbSNP (database of Single Nucleotide Polymorphism) (http://hgdownload-test.cse.ucsc.edu/goldenPath/hg19/database/), HapMap project (ftp://ftp.ncbi.nlm.nih.gov/hapmap), and 1000 Genome Project (ftp://ftp.1000genomes.ebi.ac.uk/vol1/ftp).

### Polymerase chain reaction and Sanger sequencing

All novel variants were confirmed by polymerase chain reaction (PCR) and Sanger sequencing. DNA samples of the family members (Figure 1) were taken from peripheral blood using the QIAamp DNA Blood Midi Kit (Qiagen, Hilden, Germany). A familial segregation analyses were performed by PCR and Sanger sequencing. The novel identified variants were subsequently verified and screened in the 200 healthy matched control individuals using Sanger sequencing.

### Assessment of novel mutations pathogenicity

Assessment of pathogenicity was based on the American College of Medical Genetics (ACMG) guidelines [37]. According to these guidelines, variants are categorized into pathogenic, likely pathogenic, variant of uncertain significance, likely benign, or benign. The variants obtained from ANNOVAR file were prioritized by applying a stringent filter with minor allele frequency less than or equal to 0.01% in 1000 genome, ESP, ExAC, and gnomAD [27,38].

Null variants were defined as those that were expected to introduce a premature termination codon or to affect premRNA splicing of the transcript and had been classified as pathogenic. Novel missense variants were also classified as pathogenic based on bioinformatics analysis of their pathogenicity (PolyPhen-2 and SIFT tools available online; PolyPhen-2: http://genetics.bwh.harvard.edu/pph2/; SIFT: http://sift.jcvi.org/www/SIFTenst submit.html), evolutionary conservation, and segregation with disease. A novel occurrence of large fragment deletions in the coding regions was confirmed by reverse transcription quantitative PCR (RT-qPCR).

### Statistical analysis

Data analyses were performed using SPSS version 21.0 (IBM Corp, Armonk, NY). The qPCR results were determined using a Student’s *t* test. Associations between phenotypic severity classification and genotype group classification were investigated using Fisher’s exact test. *P*-values <0.05 were considered statistically significant.

## Results

### Phenotypic determination

**Figure 2:**
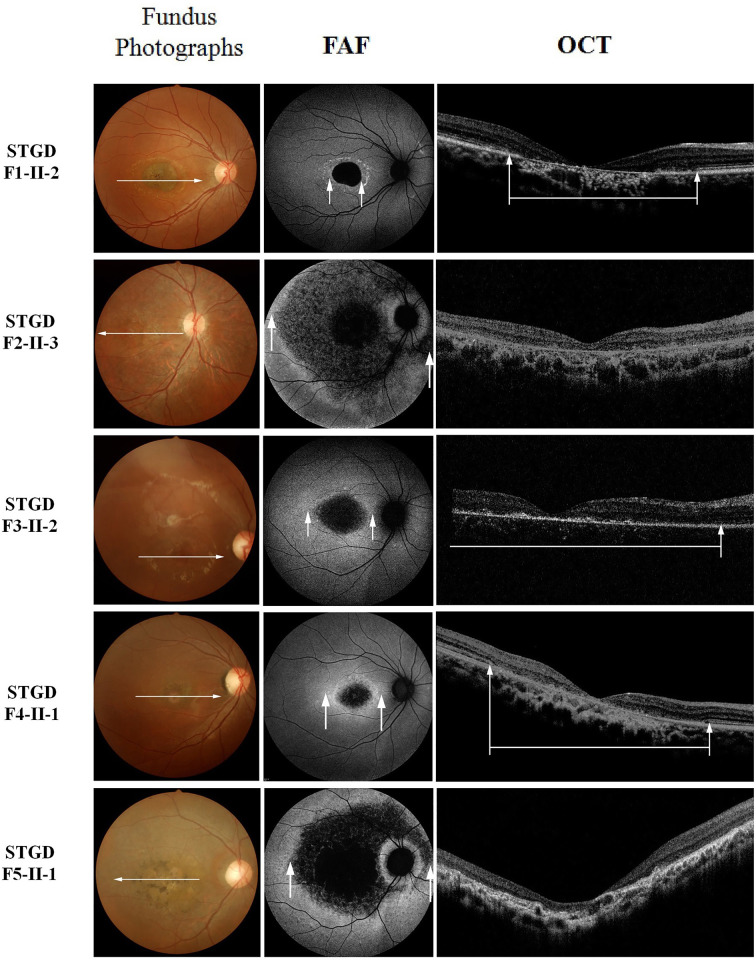
Fundus photography (left), FAF imaging (middle), and foveal OCT images (right) of the probands’ right eyes from the STGD families F1-II-2 and F4-II-1 show a mild phenotype. Localized low FAF signal is limited at the fovea surrounded by a homogeneous background.The interruption of the ellipsoid zone (EZ) band and external limiting membrane (ELM) on OCT (white arrows) corresponds to the abnormal FAF area (white arrows). F2-II-3, F3-II-2 and F5-II-1 show a severe phenotype. Multiple areas of low FAF signal extend to the vascular arcades or at the posterior pole with a heterogeneous background. Their OCT images show complete loss of the EZ band, a disorganized ELM, thinning of the outer nuclear layer, atrophy of the RPE, and choroid.

**Figure 3:**
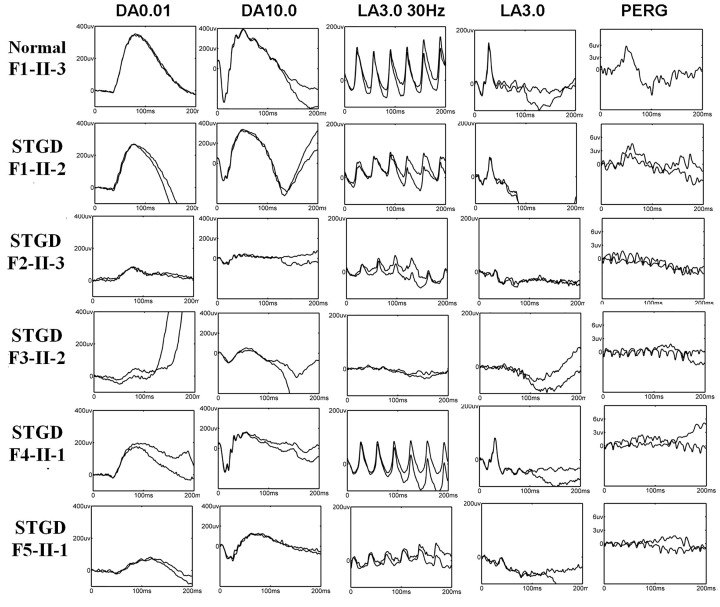
FERGs and PERGs from five representative cases of STGD harboring heterozygous *ABCA4* alleles (probands F1-II-2, F2-II-3, F3-II-2, F4-II-1, and F5-II-2) Probands F1-II-2 and F4-II-1 demonstrate undetectable PERG and normal FERGs, consistent with ERG group 1. Proband F2-II-3 has undetectable PERG, dark adapted (DA) ERGs (DA 0.01 and DA 11.0), and markedly subnormal light adapted (LA) ERGs (LA 3.0 and LA 3.0 30 Hz). Proband F3-II-2 has undetectable PERG, LA ERGs (LA 3.0 and LA 3.0 30 Hz) and markedly subnormal dark-adapted (DA) ERGs (DA 0.01 and DA 11.0). Proband F5-II-2 has undetectable PERG and markedly subnormal FERGs. The electrophysiologic findings of proband F2-II-3, F3-II-2, and F5-II-2 are classified into group 3. Normal traces are shown in the top row for comparison.

All affected patients had central visual loss, with the age of onset ranging from 3 to 20 years. They displayed a range of ophthalmological symptoms. Clinical summaries, including visual acuity, age of recruitment, gender, and relevant ophthalmological findings including fundus, FAF, OCT, ERG findings, and color vision are described in Table 1. Color fundus photographs, FAF images, and OCT images of the first five families’ probands are shown in Figure 2 (right eyes) and Supplementary Figure S1 (left eyes). Electrophysiologic traces are shown in Figure 3 (right eyes).

**Table 1:** Clinical manifestations of the proband patients with STGD

Proband	Age of consult; gender	BCVA at presentation (R/L)	Age of onset; presenting symptom	Fundus appearance	FAF type	OCT	ERG type	Phenotype severity
F1-II-2	28; F	0.25/0.30	20; a decrease in central vision	1	1	Interruption of the EZ band and ELM	1	Mild
F2-II-3	14; M	0.01/0.03	7; can not see the blackboard clearly	3	3	Loss of outer retinal layers	3	Severe
F3-II-2	9; M	0.10/0.08	3; very close TV viewing distance	2	2	Loss of EZ band and ELM	3	Severe
F4-II-1	28; F	0.20/0.15	20; a decrease in central vision	R: 1; L: 2	R: 1; L: 2	Interruption of the EZ band and ELM	1	Mild
F5-II-1	22; M	0.01/0.01	6–7; can not see the blackboard clearly	3	3	Neurosensory retina thinning and RPE atrophy	3	Severe
F6-II-1	27; M	0.06/0.06	10; a decrease in central vision	3	3	Loss of EZ band and ELM	3	Severe
F7-II-2	18; M	0.01/0.02	6–7; can not see the blackboard clearly	3	3	Neurosensory retina thinning and RPE atrophy	3	Severe
F8-II-1	9; M	0.10/0.15	5; very close TV viewing distance	2	2	Loss of EZ band and ELM	3	Severe
F9-II-1	25; M	0.20/0.20	19; a decrease in central vision	1	1	Interruption of the EZ band and ELM	1	Mild
F10-II-1	14; M	0.5/0.4	14; can not see the blackboard clearly	2	2	Interruption of the EZ band and ELM	1	Mild

Abbreviations: ELM, external limiting membrane; EZ, ellipsoid zone; F, female; L, left eye; M, male; R, right eye.

The proband in F1, F4, and F9 were adult-onset STGD patients (with a late onset of ≥17 years), and the proband in F10 was childhood-onset at 14 years, showing a relatively mild disease phenotype. Fundus photographs of the F1 and F4 probands showed typical macular atrophy of the RPE resembling a ‘beaten-bronze’ appearance, and distribution of orange-yellow flecks around the macula (fundus type 1) (Figure 2). FAF imaging demonstrated a localized low FAF signal at the fovea with a high signal edge surrounded by a homogeneous background (FAF type 1). However, the F4 proband showed high FAF signal extending anterior to the vascular arcades in the left eye (FAF type 2, Supplementary Figure S1). FAF type was bilaterally symmetric in all patients except the F4 proband in which an interocular difference in type was found (R: FAF type 1; L: FAF type 2). SD-OCT examination of this group of patients demonstrated reduced retinal thickness, disruptions of the ellipsoid zone (EZ) (also known as the inner/outer segment junction) and external limiting membrane (ELM), and atrophy of the macular RPE. The lateral edge of the lesion (white arrows) corresponded spatially with darkening of the lesion on FAF imaging (Figure 2 and Supplementary FigureS1). The relative outer retinal layer preserved beyond the edge of the lesion was seen on FAF and SD-OCT images. ERGs in this group of probands (F1, F4, F9, and F10) were consistent with ERG group 1 (Figure 3).

The patients in F2, F3, F5, F6, F7, and F8 were childhood-onset STGD patients (with an age at onset of ≤10 years in these cases). They complained of decreased central vision at the age of 3–10 years. The disease phenotypes of these patients were severe. The visual loss of F3 proband was first noted by his parents at the age of 3, when he was watching television from a particularly close distance. When seen at the age of 9, he had bulls-eye macular lesions without visible flecks (fundus type 2) (Figure 2 and Supplementary Figure S1). FAF imaging demonstrated FAF type 2 (Figure 2 and Supplementary Figure S1). SD-OCT scan identified complete loss of EZ and ELM, disruption observed mainly in outer retinal layers, neurosensory retina atrophy, and RPE thinning. The probands F2, F5, F6, and F7 showed multiple extensive atrophic changes of the RPE, extending beyond the vascular arcades (fundus type 3) (Figure 2 and Supplementary Figure S1). Moreover, the fundus photograph of the F6 proband showed extensive pigmentation in the posterior pole of the retina [36]. Their FAF and SD-OCT images were similar. FAF images showed multiple areas of low FAF signal throughout the posterior pole (type 3 FAF pattern) (Figure 2 and Supplementary Figure S1). SD-OCT identified marked outer retinal loss and generalized RPE atrophy throughout the macula. The electrophysiological findings of childhood-onset STGD (F2, F3, F5, F6, F7, and F8) probands were all classified into ERG group 3.

### Mutation analysis

Ten index patients analyzed by TES were found to be compound heterozygous or homozygous for variants in *ABCA4*, consistent with our filtering criteria (Table 2). Seventeen pathogenic mutations were identified, of which four have not been reported previously. The nonsense mutation c.6118 C > T was detected twice in F3 and F5; two families had homozygous variants (Table 2). These variants were all confirmed by Sanger sequencing.

**Table 2:** *ABCA4* mutations identified by next-generation sequencing analyses

Family	Proband	Phenotype	Gene	Exon or intron	Nucleotide change	Amino acid change	Mutation type^1^	SIFT prediction	SIFT Score	PolyPhen-2 prediction	PolyPhen-2 score	ACMG classification	Reported or novel
F1	II -2	STGD	*ABCA4*	EX45	c.6190G>A	p.Ala2064Thr	Missense; het	Damaging	0.016	Benign	0.26	Pathogenic	Zaneveld (2015, PMID: 25474345); Hu (2019, PMID: 31543898)
			*ABCA4*	IN13	c.1937+1G>A	_	Splicing; het	N.P	N.P	N.P	N.P	Pathogenic	Eandi (2014, PMID: 24585425); Xie (2020, PMID: 32619247)
F2	II -3	STGD	*ABCA4*	EX16	c.2424C>G	p.Tyr808Ter	Nonsense; het	N.P	N.P	N.P	N.P	Pathogenic	Zhou (2014, PMID: 24632595); Jiang (2016, PMID: 26780318); Liu (2020, PMID: 32845068); Hu (2020, PMID: 2678031833129279); Zaneveld (2015, PMID: 25474345)
			*ABCA4*	IN12	c.1761-2A>G	_	Splicing;	N.P	N.P	N.P	N.P	Pathogenic	Jiang (2016, PMID: 26780318)
F3	II -2	STGD	*ABCA4*	EX4	c.371delG	p.Arg124LeuTer30	Deletion;	N.P	N.P	N.P	N.P	Pathogenic	Novel
			*ABCA4*	EX44	c.6118C>T	p.Arg2040Ter	Nonsense; het	N.P	N.P	N.P	N.P	Pathogenic	Rosenberg (2007, PMID: 17982420)
F4	II -1	STGD	*ABCA4*	EX44	c.6118C>T	p.Arg2040Ter	Nonsense; het	N.P	N.P	N.P	N.P	Pathogenic	Rosenberg (2007, PMID: 17982420)
			*ABCA4*	EX23	c.3385C>T	p.Arg1129Cys	Missense; het	Damaging	0	Probably damaging	0.998	Pathogenic	Shroyer (2001, PMID: 11384574); Lewis (1999, PMID: 9973280)
F5	II -1	STGD	*ABCA4*	EX37	EX37del	_	Deletion;	N.P	N.P	N.P	N.P	Pathogenic	Novel
			*ABCA4*	EX30	c.4537_4538insC	p.Gln1513ProTer41	Insertion; het	N.P	N.P	N.P	N.P	Pathogenic	Fujinami (2013, PMID: 23499370); Chacon et al. (2013, PMID: 23419329)
F6	II -6	STGD	*ABCA4*	EX13	c.1924T>A	p.Phe642Ile	Missense; hom	Damaging	0.03	Benign	0.278	Pathogenic	Jin (2014, PMID: 24791140)
F7	II -2	STGD	*ABCA4*	IN49	c.6816+1G>A	_	Splicing; hom	N.P	N.P	N.P	N.P	Pathogenic	Xin (2015, PMID: 26161775)
F8	II -1	STGD	*ABCA4*	EX37	c.5300T>C	p.Leu1767Pro	Missense; het	Damaging	0.001	Probably damaging	0.998	Pathogenic	Stenirri (2008, PMID: 18652558)
			*ABCA4*	EX49	c.6788G>A	p.Arg2263Gln	Missense; het	Damaging	0.011	Possibly damaging	0.77	Pathogenic	Webster (2001, PMID: 11328725)
F9	II -1	STGD	*ABCA4*	EX42	c.5882G>A	p.Gly1961Glu	Missense; het	Damaging	0.001	Benign	0.234	Pathogenic	Runhart (2020, PMID: 32815999); Klufas (2018, PMID: 28248825); Lee (2017, PMID: 28327576)
			*ABCA4*	EX6	c.681T>G	p.Tyr227Ter	Nonsense; het	N.P	N.P	N.P	N.P	Pathogenic	Novel
F10	II -1	STGD	*ABCA4*	EX48	c.6563T>C	p.Phe2188Ser	Missense; het	Damaging	0.049	Benign	0.267	Pathogenic	Jiang (2016, PMID: 26780318); Hu (2019) Hu (2019, PMID: 31543898); Liu (2020, PMID: 32845068)
			*ABCA4*	EX39	c.5509C>T	p.Pro1837Ser	Missense; het	Damaging	0.003	Probably damaging	1	Pathogenic	Novel

**Figure 4:**
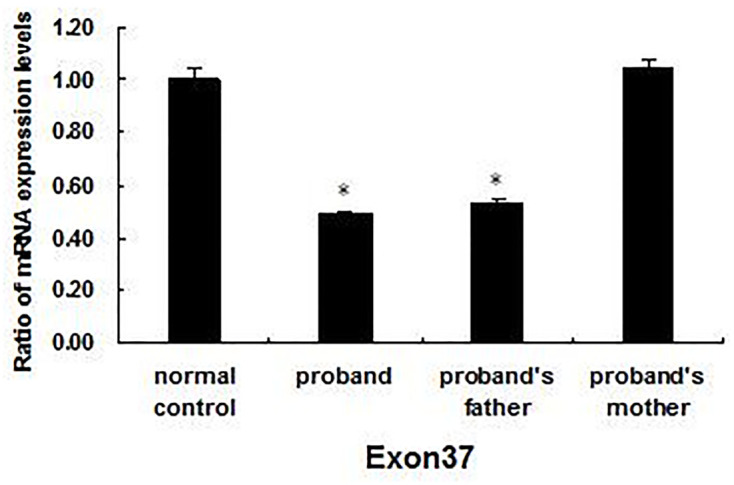
RT-qPCR results for the 37 exon in *ABCA4* The qPCR results indicated that the mRNA expression levels of the 37 exons in the proband and his father were significantly lower than those of the normal controls (*P<*0.05), but no significant difference was found between the proband’s mother and the normal controls (*P>*0.05). *P<0.05, statistical significance was determined using a Student’s *t* test.

Two of the four mutations that have not been described previously (c.681 T > G and c.371delG) were of the truncated type, predicted to create a premature stop codon, strongly indicating the loss of normal protein product and function. Another of these four was EX37del, a large fragment deletion mutation, confirmed by RT-qPCR (Figure 4). The remaining missense mutation (c.5509 C > T) was predicted to be pathogenic based on the ACMG guidelines and we verified that it met the relevant criteria. All of these mutations co-segregated perfectly with the disease phenotypes in the respective families (Figure 1) according to a recessive pattern of inheritance, as ascertained by PCR and Sanger sequencing.

### Genotype–phenotype association

In our study, the childhood-onset Stargardt (F2, F3, F5, F6, F7, and F8) appears to be a severe subtype of STGD that is characterized by early foveal abnormalities, the severe loss of visual function and generalized cone and rod dystrophy with ERG abnormalities. In contrast, in late-onset STGD (F1, F4, and F9), visual acuity and cone and rod function (ERG amplitudes) are often preserved to a relatively advanced age. Moreover, the childhood-onset Stargardt lies mostly at the severe end of the spectrum of *ABCA4*-associated genotypes. The number of childhood-onset Stargardt families in genotype Class A, B and C was four, zero, and two respectively (100, 0, 66.7%). However, F6 in genotype Class C consisted of homozygous variants. The number of the adult-onset STGD in genotype Class A, B, and C was zero, three, and one respectively (0, 100, 33.3%). A statistically significant association between phenotypic severity and genotype group was revealed (*P*<0.05). We found six studies illustrating the phenotypic/genotypic spectrum in Chinese, American and European STGD, their findings are shown in Supplementary Table S3 for comparison with the present results.

## Discussion

We successfully identified 17 *ABCA4* mutations associated with STGD in ten Chinese families and detailed their genetic and clinical characteristics. Of these 17 mutations, 4 novel and pathogenic alleles were identified: c.371delG, c.681 T>G, c.5509 C>T, and EX37del. We found that childhood-onset STGD patients were associated with severe visual loss, generalized retinal dysfunction, and had more severe variants of *ABCA4* than those found in adult-onset disease.

STGD exhibits extensive clinical heterogeneity. Its classical phenotype features yellowish-white fundus flecks and macular atrophy, but the fundus appearance can be variable: normal fundus, only flecks without macular atrophy, macular atrophy without flecks, multiple extensive RPE atrophy, and flecks and/or atrophy at a variety of locations. Most of our patients showed typical macular dystrophies (bullseye sign), but the fundus photograph of the F6 proband showed multiple pigmentations similar to retinitis pigmentosa [36]. The patients’ FAF, SD-OCT and ERG results, disease stages, and disease progression were diverse and were closely associated with each other. Our findings indicated the EZ band interruption regions in SD-OCT results were corresponding to the location of abnormal FAF, consistent with the clinical results of previous reports and also in retinitis pigmentosa [4,14,39]. The visibility of the EZ band seen on the SD-OCT, has been attributed to the scattering of light by mitochondria in the ellipsoid EZ of photoreceptor inner segments. Its disappearance has thus been an indicator of photoreceptor loss and an explanation for a decline in visual acuity [4]. The ERG results (indicators of retinal function) were closely associated with the structural changes determined by FAF and SD-OCT.

We also demonstrated that childhood-onset STGD could be considered a distinct, severe subtype characterized by a rapid loss of visual function and generalized rod and cone system dysfunction (ERG group 3). In contrast, in adult-onset STGD, visual acuity was well preserved and retinal dysfunction was limited to the macula (ERG group 1). This provides further evidence of childhood-onset STGD having a more severe retinal phenotype than adult-onset STGD, and is consistent with previous reports [14,15,20,21].

STGD exhibits extensive genetic heterogeneity. *ABCA4* encodes the retinal-specific ATP-binding cassette transporter (ABCR). The accumulation of all-trans-retinal and its toxic derivatives eventually results in the death of RPE cells and photoreceptor cells [20]. In general, null variants including PTC, premRNA splicing mutations and large fragment deletions were found to cause more severe functional effects than missense variants. Interestingly, in our study four childhood-onset STGD families (F2, F3, F5, and F7) had more severe variants (harboring two deleterious null variants) in *ABCA4* than the three adult-onset STGD families (F1, F4, and F9: harboring one null variant). These results are consistent with previous reports that individuals with truncation mutations in both of their alleles likely represent complete loss of *ABCR* function and the corresponding phenotype is reportedly younger onset and more severe disease (Supplementary Table S3) [20,28,30,40]. However, less clear agreement exists in the classification of Childhood-Onset and Adult-Onset STGD based on the age of onset [28,30]. Two childhood-onset STGD families with homozygous (F6) or heterozygous missense (F8) variants showed severe phenotypes. Perhaps some missense variants are similar to nulls, suggesting complete abrogation of protein function, and the prediction of mutant allele severity was based on *in vitro* biochemical analyses and *in vivo* studies. Alternatively, perhaps environmental and genetic modifiers contribute to the phenotypic variation [13,40,41].

Data from the four previous studies on the phenotypic/genotypic spectrum in Chinese STGD (Supplementary Table S3) indicate that the most prevalent three variants were c.101_106del (30 alleles); c.6563 T > G (23 alleles); and c.2424 C > G (19 alleles) [27,28,30,31]. The nonsense mutation c.2424 C > G p.Tyr808Ter was first identified in two affected siblings in a Chinese STGD family [7], and reported the most frequent mutation mutation, with an allele frequency 4.7% (15/322) in Chinese patients [28]. Clinical features in two siblings were severe and highly similar to those of patients in our study: early-onset (≤10 years of age), markedly decreased visual acuity, and extensive atrophic-appearing changes of the RPE to the midperipheral retina [7]. The above mutation was also found in 2 out of 31 Chinese patients with STGD [18], was the fourth most prevalent variant in a large western Chinese STGD1 cohort [30], and to our knowledge, has never been reported in patients of any other ethnic background. Therefore, we speculate that this may be a mutation specific to patients of Chinese ethnicity. The mutation c.5882 G > A was the most prevalent variant in the ProgStar study [24], and was associated with mild disease phenotype [5]. In agreement with this, in our study a patient with this mutation showed mild phenotypic characteristics.

The small sample of our study is a potential limitation which may have introduced bias to the analysis of genotype–phenotype associations. Further research is necessary to allow longitudinal observation in a wider mutation spectrum to further understand the underlying pathogenic mechanisms. Despite the limitations, we successfully identified 17 disease-causing *ABCA4* mutations, including four novel mutations using TES-based NGS in ten independent STGD families. Our findings expand the spectrum of known mutations and their related clinical phenotypes in Chinese STGD patients. A thorough clinical and genetic analysis is important for patient counseling and for ongoing stem cell or gene therapy.

## Supplementary Material

Supplementary Figure S1 and Tables S1-S3

## Data Availability

All data generated or analyzed during the present study are included in this published article and its supplementary information files.

## Abbreviations

ABCR: retinal-specific ATP-binding cassette transporter
ACMG: American College of Medical Genetics
BCVA: best-corrected visual acuity
ELM: external limiting membrane
EZ: ellipsoid zone
FAF: fundus autofluorescence
FERG: full-field electroretinography
NCBI: National Center for Biotechnology Information
NGS: next-generation sequencing
OCT: optical coherence tomography
PCR: polymerase chain reaction
PERG: pattern electroretinography
RPE: retinal pigment epithelium
RT-qPCR: reverse transcription quantitative PCR
SD-OCT: spectral-domain optical coherence tomography
STGD: Stargardt disease
TES: targeted exome sequencing
