# Supplementary material for: Targeted next-generation sequencing identifies ABCA4 mutations in Chinese families with childhood-onset and adult-onset Stargardt disease
Source: Biosci Rep. 2021 Jun 2;41(6):BSR20203497. doi: 10.1042/BSR20203497 (PMC8173525; doi:10.1042/BSR20203497)
Supplement: Supplementary Figure S1 and Tables S1-S3 [file BSR-2020-3497_supp.pdf]

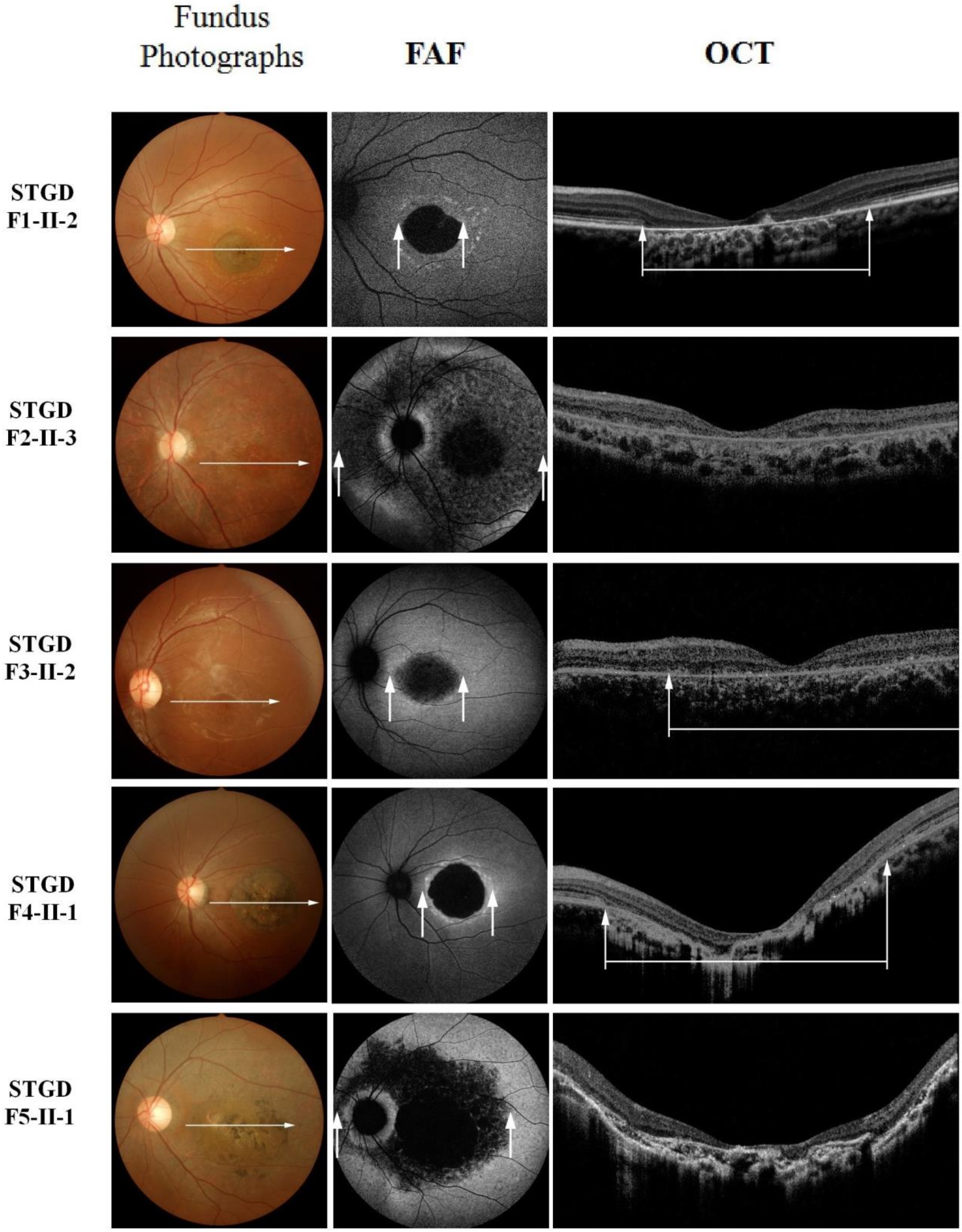
Fundus Photographs
FAF
OCT
STGD F1-II-2
STGD F2-II-3
STGD F3-II-2
STGD F4-II-1
STGD F5-II-1

Figure S1. Fundus photography (left), fundus autofluorescence (FAF) imaging (middle), and foveal optical coherence tomography (OCT) images (right) of the probands' left eyes from the Stargardt disease (STGD) families. The patients show similar classification of clinical phenotype bilaterally based on Fundus photography, FAF and OCT, with the exception of the proband F4-II-1 with different FAF types (R: type 1, L: type 1) between eyes.

Table S1 Classification of Phenotype and Genotype in Stargardt Disease, Based on Fundus Appearance, Autofluorescence (FAF) Pattern, Electrophysiologic (ERG) Assessment, and *ABCA4* Variants

Fundus Appearance		FAF Pattern		ERG group		Genotype Group Classification
Type 1	Central atrophic-appearing macular lesion with or without flecks	Type 1	Localized low AF signal at the fovea surrounded by a homogeneous background with or without perifoveal foci of high or low signal	Group 1	PERG abnormality with normal full-field ERGs	Class A:two or more severe/null variants
Type 2	An atrophic foveal/macular lesion and numerous flecks, extending anteriorly to the vascular arcades and nasally to the optic disc	Type 2	Localized low AF signal at the macula surrounded by a heterogeneous background and widespread foci of a high or low AF signal extending anterior to the vascular arcades	Group 2	PERG abnormality with additional generalized cone abnormality	Class B: One null variant
Type 3	Multiple extensive atrophic changes of the retinal pigment epithelium (RPE), extending beyond the vascular arcades	Type 3	Multiple areas of low AF signal at the posterior pole with a heterogeneous background with or without foci of a high or low AF signal	Group 3	PERG abnormality with additional generalized cone and rod abnormality	Class C: Two or more missense variants

Table S2. Classification of Severity for the Phenotype of Stargardt Disease

	Onset of Disease (yrs)	BCVA in the better eye	Fundus Appearance	FAF Type	ERG Group
Mild phenotype	Adult onset (≥17)	≥ 0.15	1	1	1
Moderate phenotype	Patients who did not meet at least 2 criteria of either mild phenotype or severe phenotype were classified into the moderate phenotype subgroup.				
Severe phenotype	Childhood onset (<17)	≤0.10	3	3	3

BCVA, best corrected visual acuity; FAF, fundus autofluorescence; ERG, electroretinograms.

For the purpose of the present study, patients who met at least 2 criteria of mild phenotype were classified into the mild phenotype subgroup and those who had at least 2 features of severe phenotype were classified into the severe phenotype subgroup.

Table S3. Prevalent *ABCA4* variants and Genotype-Phenotype Association in Chinese population

Reports	Number of affected Subjects	Recruitment criteria	Prevalent variants				Genotype-Phenotype Association
			Variant 1	Variant 2	Variant 3	Variant 4	
Xin W et al. Identification of Genetic Defects in 33 Probands with Stargardt Disease by WES-Based Bioinformatics Gene Panel Analysis. PLoS One 2015; 10(7):e0132635.	33	33 unrelated patients with STGD1 in China	c.101_106del, p.Ser34_Leu35 del (3 alleles)	c.4773+1G＞T (3 alleles)	c.5646G＞A, p.Met1882Ile (3 alleles)	c.1804C＞T, p.Arg602Trp (2 alleles)	The genotype-phenotype association was not elaborated.
Jiang F et al. Screening of ABCA4 Gene in a Chinese Cohort With Stargardt Disease or Cone-Rod Dystrophy With a Report on 85 Novel Mutations. Invest Ophthalmol Vis Sci 2016; 57(1):145-52.	161	161 unrelated patients with STGD1(96 patients)and cone-rod dystrophy (65 patients) in China	c.2424C＞G, p.Tyr808 Ter (15 alleles)	c.6563 T＞G,p.Phe2188 Ser (12 alleles)	c.101_106del, p.Ser34_Leu35del (10 alleles)	c.2894A＞G, p.Asn965 Ser (10 alleles)	For the patients in group 1, whose disease onset ages were between 1 and 10 years, the fraction (45.3%, 19/42) of patients carrying compound heterozygous or homozygous deleterious mutations was much higher than those observed for the patients in the group 2(24.1%, 7/29) and group 3 (15.4%, 2/13), whose disease onset ages were from 11 to 20 years or older than 20 years,respectively. The percentage of patients carrying compound heterozygous or homozygous missense mutations was higher in group 3 than in group 1 or group 2.
Hu FY et al. ABCA4 Gene Screening in a Chinese Cohort With Stargardt Disease: Identification of 37 Novel Variants. Frontiers in genetics 2019; 10:773.	153	153 patients from 96 unrealated families with STGD1 in China	c.101_106del, p.Ser34_Leu35 del (17 alleles)	c.2894A ＞ G, p.Asn965 Ser (10 alleles)	c.6563 T＞G, p.Phe2188 Ser (7alleles)	c.1819 G ＞ A,p.Gly607Arg(6 alleles)	Detailed genetic characteristics were illustrated. However, the comprehensive phenotypic features of the patients and genotype-phenotype association were not elaborated.
Liu X et al. Clinical and genetic characteristics of	42	42 unrelated patients with	c.1761-2A ＞ G (6 alleles)	c.2894A ＞ G, p.Asn965 Ser	c.1222C＞G, p.Arg408 Ter	c.6563 T＞G, p.Phe2188 Ser	They divided pathogenic or likely pathogenic variants into three genotype

Stargardt disease in a large Western China cohort: Report 1. American journal of medical genetics Part C, Seminars in medical genetics. 2020; 184(3):694-707.		STGD1 in China		(5 alleles)	(4 alleles)	(3 alleles); c.2424C ＞ G, p.Tyr808 Ter (3 alleles); c.53 G ＞ C, p.Arg18 Pro(3 alleles)	groups based on the presence of deleterious variants. A statistically significant association between phenotypic severity and genotype group was revealed (p <0 .05).
Fujinami K et al. Detailed genetic characteristics of an international large cohort of patients with Stargardt disease: ProgStar study report 8. The British journal of ophthalmology. 2019; 103(3):390-397.	345	345 unrelated probands with STGD1 in the USA and Europe	c.5882G>A, p.Gly1961Glu (15.05%)	c.2588G>C, p.Gly863Ala (7.17%)	c.5461 － 10 T > C (4.84%)	c.4139C>T, p.Pro1380Leu (3.94%)	There were 279 patients with multiple (at least two) pathogenic variants: group A=only severe/null variants (n=16); group B=one severe/null variant (n=124) and group C=two or more missense (n=139). Approximately half of the cohort harbours missense variants only, indicating a relatively mild phenotype of the ProgStar cohort.
Cremers et al. Clinical spectrum, genetic complexity and therapeutic approaches for retinal disease caused by ABCA4 mutations. Progress in retinal and eye research 2020; 79:100861.	N/A	STGD1 patients in numerous clinical studies in the world	The fouder variants in African American: c.6320G>A, p.Arg2107His; c.2966T>C, p.Val989Ala; c.2971G>C, p.(Gly991Arg)	The fouder variant in Western Europe :c.2588G>C, p.Gly863Ala; c.5603A>T, P.Asn1868Ile	The fouder variant in Danish : c.2894A ＞ G, p.Asn965 Ser	The fouder variant in Somali : c.5882G>A, p.Gly1961Glu	Different combinations of “mild”, “moderate”, and “severe” *ABCA4* mutant alleles were suggested to result in distinct phenotypes. Most of the *ABCA4* variants (61% of total and 50% of unique alleles) are missense mutations. The relatively low contribution of protein truncating mutations (23% of total and 33% of unique alleles) probably can be explained through the genotype phenotype correlation model in which all ABCA4-associated retinopathy cases, except those with early-onset disease, carry at least one non-truncating mutation.

N/A : data not available.
